# Severe Asthma and Biological Therapies: Now and the Future

**DOI:** 10.3390/jcm12185846

**Published:** 2023-09-08

**Authors:** Olaia Sardon-Prado, Carolina Diaz-Garcia, Paula Corcuera-Elosegui, Javier Korta-Murua, Jose Valverde-Molina, Manuel Sanchez-Solis

**Affiliations:** 1 Division of Paediatric Respiratory Medicine, Donostia University Hospital, 20014 San Sebastián, Spain; olaia.sardonprado@osakidetza.eus (O.S.-P.); paucorcuera@hotmail.com (P.C.-E.); josejavier.kortamurua@osakidetza.eus (J.K.-M.); 2 Department of Pediatrics, University of the Basque Country (UPV/EHU), 20014 Leioa, Spain; 3 Paediatric Pulmonology and Allergy Unit, Santa Lucia General University Hospital, 30202 Cartagena, Spain; carodiaz68@yahoo.es; 4 Department of Paediatrics, Santa Lucía General University Hospital, 30202 Cartagena, Spain; 5 IMIB Biomedical Research Institute, 20120 Murcia, Spain; msolis@um.es; 6 Department of Pediatrics, University of Murcia, 20120 Murcia, Spain; 7 Paediatric Allergy and Pulmonology Units, Virgen de la Arrixaca University Children’s Hospital, 20120 Murcia, Spain

**Keywords:** severe asthma, biologic, phenotype, biomarkers, omalizumab, mepolizumab, benralizumab, dupilumab, 1ezepelumab

## Abstract

Recognition of phenotypic variability in pediatric asthma allows for a more personalized therapeutic approach. Knowledge of the underlying pathophysiological and molecular mechanisms (endotypes) of corresponding biomarkers and new treatments enables this strategy to progress. Biologic therapies for children with severe asthma are becoming more relevant in this sense. The T2 phenotype is the most prevalent in childhood and adolescence, and non-T2 phenotypes are usually rare. This document aims to review the mechanism of action, efficacy, and potential predictive and monitoring biomarkers of biological drugs, focusing on the pediatric population. The drugs currently available are omalizumab, mepolizumab, benralizumab, dupilumab, and 1ezepelumab, with some differences in administrative approval prescription criteria between the U.S. Food and Drug Administration (FDA) and the European Medicines Agency (EMA). Previously, we described the characteristics of severe asthma in children and its diagnostic and therapeutic management.

## 1. Introduction

According to published studies, the prevalence of severe asthma (SA) ranges from 2% to 10% among asthmatic children [1,2,3,4]. Despite representing a small percentage of total asthmatic patients, SA-attributable costs are higher than those related to mild or moderate asthma [5]. In this way, children with SA, especially those with uncontrolled asthma (UCSA), have frequent severe exacerbations that have been associated with impairment of lung function, poor quality of life, or high risk medication side effects and interactions [6,7]. These factors not only contribute to an increase in costs, but also to an increase in unscheduled visits, emergency room visits, hospitalization admissions, school absences, and days missed from work due to caregiving [8]. What is more, these patients will have and increased risk of chronic obstructive pulmonary disease (COPD) development [9,10,11].

Based on the previously exposed, preventing asthma exacerbations as well as an adequate SA treatment is a priority objective in these children. 

### 1.1. Definition of Severe Asthma

Due to its heterogeneity, there is no universally accepted definition for SA in children. Both, the Global Initiative for Asthma (GINA) [12] and the Spanish Asthma Guidelines (GEMA) [13] establish asthma severity level according to the minimal maintenance treatment needed to control symptoms and exacerbations. Despite minimal differences between both guidelines, for individuals over six years old, SA can be established when maintenance treatment includes high doses of inhaled glucocorticoids (ICS) plus a second controller [14,15]. On the contrary, at infant and preschool age, establishment of severity could be difficult as clinical presentation of asthma often alternates recurrent exacerbation associated with respiratory infections with asymptomatic or well-controlled periods [16,17].

**Figure 1:**
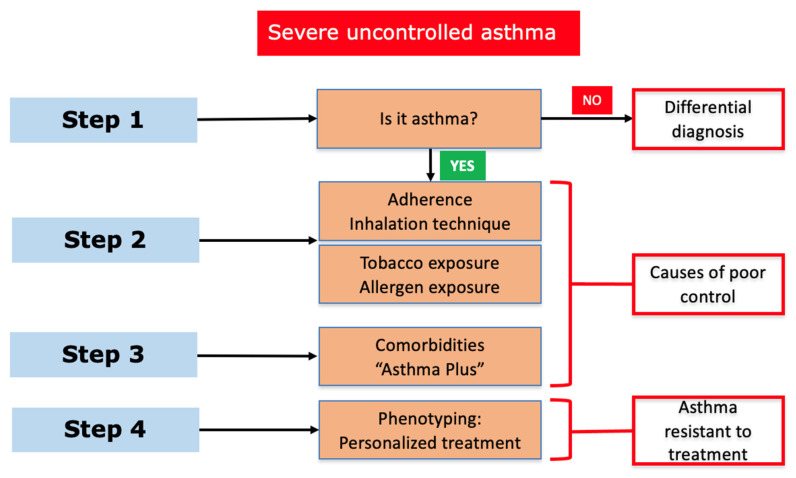
Severe uncontrolled asthma in children: stepwise assessment (GEMA 5.3).

The diagnosis of UCSA (also defined as asthma resistant to treatments) requires a multidisciplinary evaluation in order to exclude other clinical disorders with similar symptoms. In this process, it is also crucial to distinguish between UCSA and difficult-to-trat SA (related to incorrect diagnosis, presence of comorbidities, poor treatment adherence, or environmental and psychological factors). Apart of objective diagnostic techniques, such as bronchodilator or bronchoprovocation tests [18], a flowchart has been proposed by the GEMA guidelines for guiding UCSA clinical assessment [13] (Figure 1).

Asthmatic children classified as UCSA should be referred to a specialized consult. Their clinical study should include: (1) pre- and post-bronchodilator spirometry (oscillometry or plethysmography, if available), (2) inflammation marker determination (including fractional exhaled nitric oxide (FeNO) quantification, induced sputum analysis, or bronchoalveolar lavage if fibrobronchoscopy is performed); and (3) atopy study (prick test and total and specific IgE determination). Other techniques, such as fibrobronchoscopy or imaging studies (for instance, high-resolution computed tomography), are reserved for selected cases, mainly for the assessment of comorbidities and other diseases [19].

Among the most frequently comorbidities, obesity, allergic rhinitis, dysfunctional breathing, or psychological problems are included. They can significantly contribute to worsening asthma control if their management is not included as a global treatment.

Apart of comorbidities, other factors known as potentially modifiable factors also contribute towards poor asthma control. In this regard, treatment adherence, drug inhalation technique, or aeroallergens and/or environmental pollutant exposure should be taken into account [20].

### 1.2. When a Biological Treatment Should Be Considered

After potentially modifiable factor identification and control (when possible), if asthma symptoms are still poorly controlled, escalation of the treatment level should be considered. The aim of treatment escalation should lead to the prevention of exacerbations as well as to improve pulmonary function, minimizing the side effects of drugs, particularly glucocorticoids. In this way, including monoclonal antibodies (mAb) (also known as biological treatments) provides an opportunity to reduce corticosteroid dosage. Before considering mAb indication, reevaluation of the aforementioned (modifiable) factors, comorbidities, and other psychosocial factors should be reconsidered. Many patients with apparently diagnosed UCSA can significantly improve their response to treatment when these items are controlled. In order to choose the optimal pharmacological strategy, an evaluation of clinical and inflammatory phenotypes is crucial [21,22]. 

The most universally accepted recommendations regarding the use of mAbs in pediatric asthma can be find at step 5 (GINA) [12] or step 6 (GEMA 5.3) [13]. According to these guidelines, mAbs could be combined with high-dose ICS plus a long-acting β2-agonist (LABA), as well as with other drug such as tiotropium, leukotriene receptor antagonist, azithromycin, and/or oral corticosteroids.

## 2. Phenotypes and Biomarkers

### 2.1. Phenotypes

Asthma phenotype is an observable characteristic that can be associated with an underlying mechanism called an endotype. Establishing a phenotype in patients with SA is critical, as it may lead to a different clinical treatment, carrying future implications [23].

The primary phenotype classifications were based on different factors, which included clinical presentation, age, or time course. Current asthma phenotype classification is based on the inflammatory pattern, or the predominant type of bronchial cellularity. For its assessment, biostatistical cluster analysis of different variables (pathobiology, pulmonary function, clinical characteristics, or biomarkers, among others) has been used [24]. According to the current evidence, identifying phenotypes based on the underlying inflammatory pattern could be the most promising strategy for targeting asthma treatments. 

Two major inflammatory phenotypes have been defined [25]: T2 phenotype (which includes allergic and eosinophilic asthma) and non-T2 phenotypes. With regard to the clinical and therapeutic approach to SA, considering three different phenotypes seems to be more appropriate: (1) the allergic-T2 phenotype, (2) the eosinophilic-T2 phenotype, and (3) the non-T2 phenotype. In the pediatric population, phenotype 1 seems to be the most frequently described, followed by 2. Phenotype 3 is much less frequent at this age [26].

On one hand, the allergic-T2 phenotype usually involves polysensitization to different allergens, in association with other atopic comorbidities. Immunologically, it is defined by a T2 inflammatory response (elevated IgE, peripheral eosinophilia, and elevated exhaled nitric oxide fraction) [26]. On the other hand, the non-allergic eosinophilic phenotype seems to be associated with chronic rhinosinusitis and nasal polyps (more frequent in adult patients) without allergen sensitization. Pathogenically, alterations in arachidonic acid metabolism are implicated, resulting in an IL-5-mediated eosinophilic inflammation of the airway. This kind of inflammation will be refractory to high doses of corticosteroid. Although prevalence of atopy is low in this phenotype, IgE and FeNO may be also elevated [26]. The non-T2 phenotype is often defined by a paucigranulocytic profile, with normal levels of eosinophils in the airway, low FeNO levels, peripheric neutrophilia, and poor response to glucocorticoids [26]. 

The Asthma Phenotypes in the Inner City (APIC) longitudinal study has identified up to five phenotypic clusters, two of them severe [27]. It defines a first cluster with high levels of T2 biomarkers, frequent symptoms, impaired lung function, and frequent exacerbations, being refractory to high doses of conventional therapies. These children could be candidates for biological therapies, including mAbs, against T2 inflammation mediators. It also defines a second cluster population with low or absent levels of T2 biomarkers that are clinically very symptomatic with moderate lung function impairment and frequent exacerbations, despite being treated with conventional therapies. Unlike the previous cluster, this group is heterogeneous, with different underlying mechanisms and variables that interact in its pathogenesis. For this reason, more research will be needed in order to clarify a specific treatment for these patients.

Based on the previously exposed explanation is the challenge of phenotyping children with SA. However, the interpretation of lower respiratory tract samples should be carefully done, especially if they are under treatment [21].

Beyond phenotypes, an attempt for a better understanding of asthma through patients’ classification into endotypes has also be performed. Endotypes can be defined as different subtypes of asthma based on a single pathophysiological mechanism. They are characterized by different biomarkers and different responses to treatment. It has to be considered that the same endotype could include different phenotypes. However, the development of new analysis techniques has increased the interest in a new concept: molecular phenotyping of asthma. In this regard, a recent trial of mepolizumab in children and adolescents was published [28]. According to this study, the upper airway transcriptome profile is a better predictor of mepolizumab efficacy than conventional biomarkers such as blood eosinophils or FeNO. These findings highlight the relevance of including molecular phenotyping methods to advance the field of targeted and personalized treatment.

### 2.2. Biomarkers

The inclusion of biomarker analysis has taken a crucial role in the phenotypic classification of patients with SA. It allows for concrete pathogenesis pathway identification as well as the prediction of treatment response, its monitoring, or early of disease progression. However, phenotype-/endotype-specific biomarkers are currently unavailable, with the most commonly used being the ones related to the T2 phenotype. They include the quantification of peripheral blood and sputum eosinophil concentration, FeNO measurement, and determination of serum IgE and periostin.

One of the most important biomarkers is total eosinophil count in peripheral blood. It is easy to obtain and widely used, predicting not only the risk of exacerbations in children and adults [29] but also the response to biological treatments. When peripheral blood eosinophil count is greater than 150–300/µL during the last year, a T2 phenotype can be proposed [13,30].

Airway eosinophil quantification is probably more directly related to the inflammatory phenotype of asthma; however, sampling, either by bronchoalveolar lavage or sputum, is difficult in terms of its inclusion in a routinary study of children.

Within the exhaled biomarkers, FeNO, as an indicator of IL-13-mediated inflammation, is another non-invasive marker that allows for the identification of a T2 phenotype (FeNO ≥ 25 ppb) [13,30]. Nevertheless, some limitations to its use have been described, as its determination could be influenced by age; ethnicity; or environmental factors such as diet, viral infections, or smoking [31].

Other exhaled biomarkers such as volatile organic acids (VOCs) [32], oxidative stress markers, leukotrienes, or cytokines are still under study. In fact, sampling or quantification techniques have not been standardized. With this purpose, specialized and validated laboratories would be needed but, unfortunately, their cost would make it unaffordable for daily pediatric practice [33].

Total IgE and specific IgE levels are also important as T2 inflammation biomarkers. High levels, as an indicator of allergic sensitization, are associated with asthma development and an increasing in asthma morbidity in early childhood [34]. Indeed, the presence of allergic sensitization has been associated with the degree of response to inhaled glucocorticoid treatment. Furthermore, sensitization combined with allergen exposure is an important predictor of exacerbations, morbidity, and response to anti-IgE therapy in children.

With regard to periostin, it is a cell matrix protein induced by IL-13 and IL-4 that is secreted by bronchial epithelial cells and that can be detected in the blood. Its synthesis has been associated with airway remodeling, subepithelial fibrosis, eosinophilic recruitment, and the regulation of mucus production by goblet cells. For this reason, periostin quantification could help in the identification of patients with severe uncontrolled asthma and those susceptible to anti-IL-13 therapy. However, periostin’s usefulness in childhood asthma is still unclear. Some studies have shown significantly higher values in children with asthma than in healthy controls. In the same way, a positive correlation between higher serum periostin levels and induced bronchial hyperresponsiveness (BHR) has been described. On the contrary, the relationship between blood eosinophils and IgE is still unclear. In a real-life study [35], it was shown that serum periostin was not related to the degree of asthma control in children, remaining unclear as to whether it could have a predictive value in identifying SA in the pediatric population. Despite this possible usefulness of periostin, it has to be taken into account that its determination is only readily available in some laboratories.

Evidence regarding the stability of these biomarkers in children and adolescents is limited. Longitudinal studies performed in adults suggest the need for repeated evaluations as T2 inflammation marker levels seem to vary from one determination to another [36].

Regarding the non-T2 phenotype, although neutrophils are the predominant cells in the airway, their quantification has not been defined as a marker. In fact, the quantification method makes it unattractive for routine practice, being necessary to establish cut-off points to standardize decision making. Their quantification in children’s peripherical blood is easy to perform, but it correlates poorly with counts of airway neutrophils [37].

Currently, some biomarkers are still under development and validation. Among them, YKL-40, a chitinase-like protein that has been detected in peripheral blood and in the lungs, seems to be related to asthma severity indices and risk of exacerbation. Nevertheless, in the pediatric population, it has shown a slight relationship with high FeNO levels (r = 0.48), serum neutrophilia (r = 0.63), and airway remodeling (r = 45) [38].

In the same way, urinary bromotyrosine, a biomarker resulting from eosinophil degranulation, is still under experimentation. It can be determined by a non-invasive method and has been directly related to the T2 inflammatory pathway. This marker has been associated with asthma exacerbations in children, or with monitoring glucocorticoid clinical response, especially when combined with increased FeNO and elevated sputum eosinophils [39].

## 3. Biologicals Treatments 

**Figure 2:**
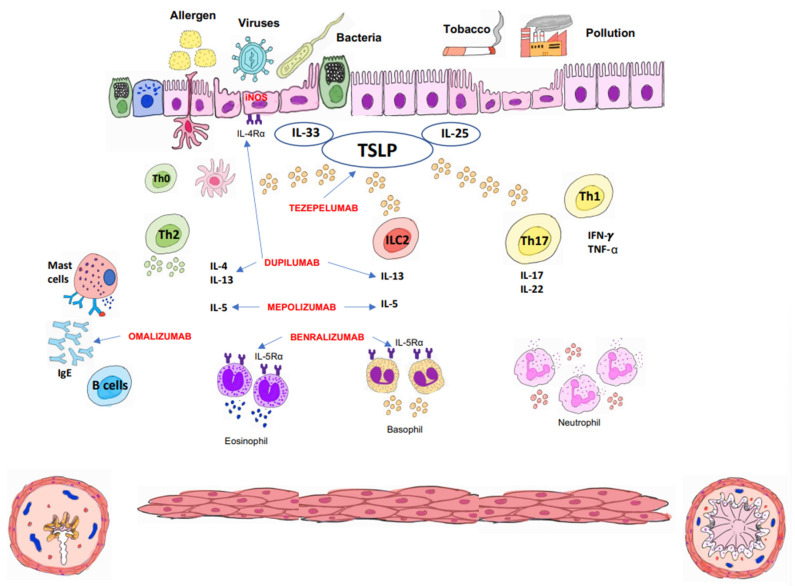
Immunopathology of asthma, biological drugs, and their therapeutic targets. TSLP: thymic stromal lymphopoietin.

Since 2014, when GINA, followed by the rest of the clinical guidelines, proposed the new recommendations for asthma clinical management, mAbs have become a key treatment for maintenance therapy in UCSA, displacing classical treatments such as corticoids. Development of mAbs for new therapeutic targets (Figure 2) in recent years has facilitated a personalized treatment of patients [22,30,40,41,42,43]. Table 1 summarizes pediatric currently available mAbs, as well as their therapeutic target, dosage, route of administration, clinical efficacy, and adverse effects. 

### 3.1. Omalizumab

Omalizumab is an anti-IgE humanized mA that binds to the Fcε3 segment (high-affinity receptor) of free IgE, preventing its binding to the FcεRI receptor at the surface of mast cells, eosinophils, and basophils. This leads to a rapid removal of the circulating IgE–mAbs complex by the endothelial reticulum system, decreasing both circulating IgE levels and receptor expression at the cell surface. It will finally result in a reduction of cytokine release at the allergic inflammatory cascade.

Omalizumab was the first biologic drug approved for treatment of asthma in children over 12 years old by the U.S. Food and Drug Administration (FDA) in 2003, as well as by the European Medicines Agency (EMA) in 2005. For children over 6 years of age, it was approved in 2016. 

To explain certain effects of this drug, it is worth recalling virus- and pneumoallergen-induced inflammation in patients older than 3 years. For instance, allergic sensitization with increased IgE levels against house dust mites may increase the likelihood that a respiratory tract infection, particularly when rhinovirus is involved, could lead to asthma exacerbation [44]. A link between IgE levels, overexpression of FcεRI, and suppression of the virus-induced plasmacytoid dendritic cell INFα response has been described. In this way, omalizumab treatment decreases FcεRI receptor expression and restores the INFα response against virus (rhinovirus and influenza) infection, contributing to the prevention of exacerbations [45].

Omalizumab has been indicated for SA patients aged ≥6 years with an allergic high-T2 phenotype who meet the following criteria: perennial pneumoallergen sensitization, increased total serum IgE (>30 and <1500 IU/mL), and chronic idiopathic urticaria. However, not all patients respond to this treatment. The following are considered as good predictors of treatment response: presenting multiple sensitizations, elevated total Ig E levels, elevated FeNO (≥20 ppb) [22], and blood eosinophilia values ≥ 300 cells/µL. Studies providing metabolomics data could help to identify a good responder profile [46]. On the contrary, age older than 12 years, exacerbations within the last 6 months, a predicted FEV_1_ <90%, or comorbidities (obesity, gastroesophageal reflux (GER), chronic rhinosinusitis, nasal polyps, and psychological disorders) can be considered as clinical predictors of poor response to treatment [47]. 

This mAb should be administered subcutaneously every 2–4 weeks, using a weight and pretreatment IgE-serum-level-based normogram for dosage calculation. Final doses will range from 75 to 600 mg (0.016 mg/kg/IgE (I.U./mL) per 4 weeks). The maximum dose is 600 mg and 375 mg every 2 weeks in Europe and the USA, respectively. Plasma half-life time is about 26 days [48]. There are 75 and 150 mg pre-filled syringes and 150 mg ampoules currently available, and they can be easily administered at home.

Regarding off-label use of omalizumab when IgE levels are higher than 1500 IU/mL, the dose calculation can be done considering a 0.016 mg/kg/IgE (I.U./mL) minimum doses every 4 weeks with a maximum dose of 600 mg every 2 weeks [49]. In the case of patients under 6 years of age, the possibility of a compassionate use treatment has been published, resulting in a significant reduction in exacerbations being documented. For this purpose, the sign of informed consent by patient’s parents or legal tutors will be mandatory [50].

Numerous studies have evaluated real-life results of omalizumab in pediatric patients, including different populations [51,52,53,54]. Among them, the most extensive is the ANCHORS study (Asthma in Children: Omalizumab in Real Life in Spain) [50], which included 426 patients during a six-year follow-up period. As was published [55,56], omalizumab treatment showed a significant decrease in exacerbations, consumption of oral and inhaled corticosteroids since the first year of treatment, and FeNO levels, as well as an increase in FEV1 and mesoexpiratory peak flow (MMEF) during the first year, or an improvement in the clinical control questionnaires of asthma punctuation.

These studies also described a progressive drop in peripheral blood eosinophil level. However, there is no global consensus on the improvement on lung capacity [55]. 

Despite current evidence, the optimal duration of treatment and long-term side effects are unknown. It is proposed that response to treatment can be assessed at 4 months, being extendable to 6 months for late responders. Therapy could be prolonged for 6 years [50] or interrupted for a period of 2 and 6 years if good control is achieved [57]. Some authors [58] have proposed its maintenance for a minimum of 18 months if the initial response has been adequate. If at that time oral corticosteroids are not required and pulmonary function is better than at the start of treatment, reducing reduction of dose by half could be considered. After 6 months, a new evaluation should be performed with the aim of progressively reducing the dose until complete withdrawal. Other authors [59] have proposed maintaining treatment for at least 2 years and evaluating discontinuation if there is no active allergic disease without exacerbations in the last year, and if FeNO levels as well as peripheral blood eosinophils decreased.

Different meta-analyses [56,60] have been published about the efficacy and safety of omalizumab. The adverse effects reported were infrequent, including headache, nonspecific symptoms (fever, myalgia, etc.), pain and/or skin reaction at the puncture site, and anaphylaxis (with a 0.2% described frequency). No increment in cancer development has been described. As SA treatment is progressively starting at younger ages, monitoring of possible adverse effects is crucial [61].

Despite omalizumab having not shown evidence on disease evolution modification, the Preventing Asthma in High-Risk Kids (PARK) study (NCT02570984) is currently underway [62]. Its aim is to determine whether treatment for 2 years in children 2–3 years of age at high risk for the development of the disease could prevent its progression and reduce its severity.

### 3.2. Mepolizumab

Mepolizumab is a recombinant humanized IgG1 mAb against IL-5, blocking its interaction with its receptor. This prevents the specific signaling cascade from being triggered. IL-5 is a potent mediator of the inflammatory cascade in the allergic response, one that binds to the α-chain of the IL-5 receptor (IL-5R). This binding modulates eosinophil maturation in the bone marrow, as well as its recruitment, alongside activation at sites of allergic inflammation. It also regulates the functions of basophils and mast cells, enhancing the release of their mediators [63]. In this way, IL-5 contributes to tissue damage, aggravating chronic airway inflammation and predisposing one to exacerbations [43]. It was approved in 2015 by the FDA and the EMA as an add-on treatment in SA for patients aged ≥18 years with an eosinophilic phenotype. In 2019, these recommendations were extended to patients aged ≥6 years [64].

Mepolizumab is an adjunctive treatment that is indicated in patients with eosinophilic UCSA who had peripheral blood eosinophil count >150 cells/µL or >300 cells/µL in the last year, as well as who, despite having been treated with the GINA step 4–5 proposed treatment [12], did not achieve an optimum control and/or presented dependence on systemic corticosteroids. It is also indicated for patients with chronic rhinosinusitis and severe nasal polyps without adequate response to systemic corticosteroids and surgery indications, as well as patients with uncontrolled hypereosinophilic syndrome without identifiable secondary cause.

The recommended dose is 100 mg every 4 weeks in children ≥12 years and 40 mg every 4 weeks in children between 6 and 11 years. It is administered subcutaneously, being easy to handle at home.

The MENSA study, which included adolescents, observed that the patients were more atopic, with a shorter disease course and a higher number of hospitalizations for exacerbations than the general population [65]. The eosinophil count, previous exacerbations, and response to treatment did not differ from that shown in adults, with a significant decrease in exacerbations and a reduction in the number of eosinophils in both the blood and sputum. Plasma concentrations, drug clearance, and safety profile were similar to those presented in adults. Treatment was not withdrawn due to adverse effects, with some of the most frequently described being headache, nasopharyngitis, and abdominal pain. Five adolescents reported severed adverse effect, but no relation to treatment was finally found. Some of the previously included adolescents continued for 52 extra weeks in the COSMOS study [66] in order to maintain a stable and durable drug effect over time. The safety profile was satisfactory and similar to that of the general population.

When both age groups, adults and adolescents, were analyzed, the magnitude of reduction in exacerbations was greater in those patients with higher peripheral blood eosinophil counts (≥150 cell/μL) than in those with lower counts. It seems that this is the best biomarker for predicting response to treatment [67]. On the contrary, pre-treatment FeNO values did not predict the clinical efficacy of treatment [68].

The MUPPITS-2 study performed in the USA included a much larger sample: 290 African American and Hispanic schoolchildren, aged 6 to 17 years, from socioeconomically disadvantaged urban neighborhoods. A randomized, double-blind, placebo-controlled, parallel-group trial was carried out [28]. The authors observed that mepolizumab significantly reduced the number of exacerbations while maintaining a good safety profile. No change in lung function or variation in asthma control questionnaire punctuation was obtained. Moreover, the authors concluded that there were specific gene expression patterns in nasal epithelial cells, and their identification predicted the risk of exacerbations despite treatment with mepolizumab. In this way, possible new biomarkers that could improve the management of patients with SA in a new precision medicine could be described [3].

An observational, open-label, multicenter, observational cohort study is currently under way in Spain and the United Kingdom. This study includes children aged from 6 to 17 years with a clinical diagnosis of eosinophilic SA treated with mepolizumab, with the aim of analyzing the effectiveness and safety (ClinicalTrials.gov Identifier: NCT05139381).

Regarding the duration of treatment, its long-term use could maintain a stable effect. However, the optimal duration is still unclear [63].

To study pharmacokinetics and pharmacodynamics, 36 patients aged from 6 to 12 years who met the criteria for eosinophilic SA were recruited. A 40 mg dose was administered to children weighing less than 40 kg and a 100 mg dose if the weight was greater than 40 kg [69]. The drug’s reported half-life was similar to the one described in adults, although it was appreciably longer in children (22–24 days) than in adults (16–22 days). Bioavailability was higher in children. The reduction in blood eosinophils was accompanied by an improvement in the symptom control questionnaires scores. A decrease in the incidence of exacerbations was also reported. There was no change in pulmonary function, as has been published by other authors [70]. There were no cases of anaphylaxis or neoplasm risk, or incidence of opportunistic infections. Local reactions decreased with time. Eosinophil concentration, which was initially reduced, began to increase at the end of treatment but did not reach pre-treatment values. In this sense, the relative change in peripheral blood eosinophils was not associated with treatment efficacy [28]. The safety and efficacy profile is still being studied in the extension study, where 29 patients continued with the treatment [71].

### 3.3. Benralizumab

Benralizumab is a humanized mAb IgG1κ afucosylated mAb that binds to the α-subunit of the IL-5 receptor, specifically expressed on the surface of eosinophils and basophils. Therefore, benralizumab is able to inhibit their activation, inducing a rapid and almost complete eosinophil depletion by a mechanism of cytotoxicity mediated by N.K. cells.

Its prescription is indicated for additional maintenance therapy in patients with eosinophilic SA that requires a high dose of inhaled glucocorticoids plus LABA. It has been included for individuals aged ≥12 years by the US-FDA. On the contrary, its use has not been authorized for the pediatric population by the EMA.

The recommended dose is 30 mg, injectable subcutaneously, every 4 weeks for the first 3 doses and every 8 weeks after that. There are preloaded syringes or pens available. Home administration is possible.

Two randomized, placebo-controlled, phase III clinical trials, SIROCCO [72] (48 weeks) and CALIMA [73] (56 weeks), evaluated 2511 patients aged from 12 to 75 years, with weight ≥40 kg. All of them presented UCSA, despite treatment with a high dose of inhaled glucocorticoids plus LABA. The study concluded that the addition of benralizumab every 4 to 8 weeks resulted in a significant relative reduction in the risk of severe exacerbations, from 28 to 51% in patients with ≥300 peripherical blood eosinophils/µL. A significant improvement in pre-bronchodilator FEV_1_ in clinical disease control scales and the reduction of oral corticosteroid cycles (especially in those patients with ≥150 eosinophils/µL) were also described. These results were independent of serum IgE concentrations and atopy status [74,75].

A phase III extension study of patients included in the SIROCCO and CALIMA studies, the BORA study, demonstrated that efficacy and safety were maintained during a second year of treatment [76]. This study enrolled 86 adolescents who received benralizumab every 4 (*n* = 25) or 8 (*n* = 61) weeks for an extra period of 2 years, finding similar efficacy and safety profiles [77]. Both groups had similar efficacy with a low crude annual exacerbation rate (≤0.46). However, changes in lung function were greater in adolescents treated every 8 weeks, being more pronounced in those with ≥300 eosinophils/μL. 

Among patients included in the SIROCCO and CALIMA studies, the predictors of clinical effectiveness were higher eosinophil levels (≥300 eosinophils/μL) and previous exacerbations [78]. In addition, baseline patient factors such as the use of oral corticosteroids, the coexistence of nasal polyposis, and a FVC <65%, especially in patients treated every 8 weeks and with <300 eosinophils/μL, were also predictors of response [79]. A meta-analysis confirmed that benralizumab is a safe drug, although vigilance for adverse effects in long-term treatment is needed [80].

Several studies have recently been completed in the pediatric population. An open-label phase III study to evaluate the pharmacokinetics, pharmacodynamics, and safety of benralizumab (PATH-HOME study) was performed on 30 children aged from 6 to 11 years [81]. In parallel, two studies (the MIRACLE study NCT 03186209 [82] and the study NCT01928771 [83]) are under way to assess efficacy and safety in patients with UCSA on high-dose maintenance therapy. In addition, a multicenter, randomized, double-blind, parallel-group, phase III study is underway, looking at efficacy and safety in pediatric patients with eosinophilic SA (the DOMINICA study, NCT05692180) [84].

### 3.4. Dupilumab

Dupilumab is a fully human mAb that inhibits inflammatory signaling by specifically blocking the α-subunit of the IL-4R receptor shared by both IL-4 and IL-13.

The US-FDA approved its use for treating SA in 12-year-old patients in October 2018, and the EMA did so in March 2019. It was subsequently approved by both the FDA and EMA for children aged 6 to 11 years.

On the one hand, IL-4 plays a crucial role in differentiating virgin CD4+ T cells into Th2 cells. It also drives IgE isotype switching in B cells. On the other hand, IL-13 induces the expression of the inducible nitric oxide synthase (iNOS) enzyme in epithelial cells, leading to an increase in the exhaled fraction of nitric oxide (FeNO); it causes mucosal hypersecretion and stimulates airway smooth muscle cell contraction, leading to bronchoconstriction. IL-4 and IL-13 play an important role in the recruitment of eosinophils from the blood circulation to the airway mucosa by two main mechanisms: by enhancing the expression of adhesion molecules on endothelial cells, and by inducing the production of chemokines and eotaxin by epithelial cells. Likewise, eosinophils will be recruited by eosinophil extracellular traps, Charcot–Leyden crystals, and oxidants generated by eosinophil peroxidase. Airway eosinophils mediate the formation of mucus plugs and contribute to chronic airflow obstruction [43]. Dupilumab’s blockade of IL-4 and IL-13 prevents eosinophil migration into lung tissue by inhibiting the production of eotaxin and vascular cell adhesion molecules. Nevertheless, it does not affect eosinophils in the bone marrow, where their terminal differentiation occurs. Thus, it prevents infiltration of eosinophils into lung tissue but does not affect their production or release into the bloodstream from the bone marrow. This could explain the temporary increase in the circulating eosinophil count observed in some patients [85]. 

Dupilumab is indicated for patients older than 6 years with SA and T2 inflammation who have elevated peripheral blood eosinophils (>150 cells/µL < 1500) and/or elevated FeNO (>20 ppb). Atopic dermatitis, eosinophilia > 300 cells/µL, and/or FeNO > 50 ppb is associated with a better response. It is also approved in patients with severe-to-moderate atopic dermatitis from 6 months of age, chronic rhinosinusitis with severe nasal polyposis not controlled with medical and/or surgical treatment, severe moderate nodular prurigo, and eosinophilic esophagitis in patients >12 years.

Doses vary according to the pathology to be treated [86], being administered subcutaneously. From 12 years of age, one starts with a 400 mg dose (two injections of 200 mg) to then continue with 200 mg every two weeks. If the patient is being treated with oral corticosteroids or has moderate/severe atopic dermatitis, or severe chronic rhinosinusitis with nasal polyposis, an initial 600 mg dose (two injections of 300 mg) will be administered, followed by 300 mg every two weeks. In patients aged from 6 to 11 years, the dose depends on body weight: 15 kg–<30 kg: 100 mg every two weeks or 300 mg every 4 weeks; 30 kg–<60 kg: 200 mg every two weeks or 300 mg every 4 weeks; and ≥60 kg: 200 mg every 2 weeks.

Its bioavailability is 61–64% after subcutaneous administration, and the mean time to peak serum concentration is 3–7 days [87]. Subcutaneous administration is by pen or prefilled syringe. The pen is only indicated for patients over 12 years of age and is available in 200 mg and 300 mg formats. The prefilled syringe is also available in 200 and 300 mg and can be administered at home.

Dupilumab has demonstrated its efficacy and safety profile in several studies including adults and adolescents [88,89,90]. After analyzing its effect in a meta-analysis [91,92], a significant decrease in exacerbations and asthma symptoms, as well as an increase in quality of life and FEV_1_, were observed. These results suggested a potential effect on airway remodeling. 

The response was better if eosinophilia was ≥300 cells/µL and FeNO ≥ 50 ppb [88]. The safety profile was acceptable, and the most frequent adverse effects were upper respiratory tract infections, erythema at the site of infection, and headache [93]. There was no increase in viral, bacterial, or opportunistic infections.

The post hoc analysis of the phase III LIBERTY ASTHMA QUEST study [94] evaluated the efficacy of dupilumab in adolescent patients aged from 12 to 17 years (*n* = 107). This study concluded that there was an improvement in lung function, asthma control, and quality of life. The response was greater as the higher initial increase in T2 biomarkers was described. Concerning the reduction of exacerbations, a discrepancy was observed between patients treated with a 200 mg dose (46% reduction) and those treated with a 300 mg dose, in whom there was an increase when compared with the placebo. The authors justified this finding by an imbalance in the matching, by the number of severe exacerbations presented in the last year, and between the two groups, which could have affected the adjusted rate of exacerbations. 

Similarly, the VOYAGE study [95], conducted in children aged from 6 to 12 years, demonstrated that, in patients with T2 asthma, who presented eosinophilia between >150 µL and <300 µL and/or FeNO > 20 ppb, there was a significant reduction in exacerbations, with less administration of systemic corticosteroids. In the same way, and improvement in lung function and an increment in FEV_1_ was described. The response was rapid and was maintained for at least 52 weeks. The side effects were similar to those reported in adults and adolescents, with cases of eosinophilia (blood eosinophils ≥ 3000/µL; 6.6%) reported without an association with clinical symptoms. The long-term safety profile for 104 weeks reported in the EXCURSION study [96] was in concordance with the one described in the VOYAGE study [95]. The increase in eosinophil count observed decreased or returned to baseline after the first 24 weeks of treatment. This increase was not associated with clinical symptoms or sequelae and had no observable impact on the efficacy of the treatment received. However, it is recommended that these results continue to be monitored since the studies were limited to patients with eosinophil counts below 1500 cells/µL, and the obtained results cannot be extrapolated to patients with higher eosinophil counts. The efficacy observed in blocking IL-4 and IL-13 signaling may likely be related to other known functions of both interleukins, including effects on B-cell to IgE class switching, mucus production, goblet cell hyperplasia, collagen production, and smooth muscle cell contractility [95].

### 3.5. Tezepelumab

Tezepelumab is a humanized IgG2λ mAb that binds to thymic stromal lymphopoietin (TSLP), preventing it from interacting with its heterodimeric receptor. TSLP is a cellular-epithelium-derived alarmin-group cytokine that is released in response to an allergen, viruses, and/or pollution exposure. Its function is to induce T2 and non-T2 cytokine production, with increased bronchial hyperresponsiveness, mucus hypersecretion, and airway remodeling.

It is indicated as an additional maintenance therapy in patients with UCSA, regardless of their phenotype, despite being treated with high doses of inhaled glucocorticoids in combination with other maintenance therapy medication. It has been authorized by the FDA and EMA for patients aged ≥12 years. There is also the possibility of home use. 

The recommended dose is 210 mg, injectable subcutaneously by syringe or prefilled pen every 4 weeks.

A phase III, multicenter, randomized, double-blind, placebo-controlled study (the NAVIGATOR study) included 1061 patients aged from 12 to 80 years with UCSA. As inclusion criteria, the pre-treatment with medium-high doses of ICS (≥500 μg fluticasone propionate) plus other controller medication was defined. No OCS diagnosis or pre-study biomarker levels were defined as initial variables. Patients were randomized to receive placebo or 210 mg of tezepelumab subcutaneously every 4 weeks for 52 weeks [97]. The study reported a 56% (95% CI, 47–63%) relative reduction in the global rate of severe exacerbations, as well as a 30% specific reduction in the 82 adolescents included. Although this reduction was statistically significant for all eosinophil levels considered, it was greater for levels above 150/µL (39% for values < 150/µL to 77% for values > 450/µL). Likewise, the reduction in exacerbations was greater for higher FeNO levels, going from 32% for FeNO values < 25 ppb to 73% for FeNO values >50 ppb. In addition, it improved baseline FEV_1_, degree of asthma control, and quality of life. It also decreased the number of eosinophils, FeNO levels, and serum IgE. This study included 82 adolescents with a reduction in the rate of severe exacerbations of 0.70 (95% CI, 0.34–1.46). The authors found no differences according to perennial allergen sensitization status. 

Recently, a systematic review analyzed the efficacy of different biologics treatments in UCSA, which were stratified by eosinophil levels. Only tezepelumab showed a reduction in the annualized rate of exacerbations in patients with eosinophil levels <150/µL [98]. A multicenter, randomized, double-blind, phase III extension study was performed to evaluate the safety and tolerability of tezepelumab after 2 years of follow-up in adolescents and adults with long-term UCSA (the DESTINATION study). This study included 951 participants and corroborated the results of previous studies [99]. According to the current evidence, tezepelumab is a well-tolerated and safe drug, with the most frequent adverse effects being described as local reactions at the injection site, pharyngitis, arthralgias, and lumbar pain [97,100].

A phase I study that evaluated pharmacokinetics in 18 children with asthma aged from 5 to 11 years has recently been completed (the TRAILHEAD study, NCT04673630) [101]. In addition, the HORIZON study, a multicenter, randomized, double-blind, parallel-group, placebo-controlled, phase III study, which includes children with UCSA aged 5 to 11 years, with the main objective of analyzing tezepelumab efficacy and safety, will soon begin.

## 4. Personalized Treatment

The choice of the most appropriate biological drug for a given patient in daily clinical practice remains a significant challenge in the pediatric population. The selection is based on a specific assessment of uncontrolled asthma-contributing factors. It includes, among others, the determination of some biomarkers, such as peripheral blood eosinophil count, exhaled fraction of nitric oxide (FeNO), allergic sensitization, or IgE levels [22]. There is a clear need for further studies on biomarkers to support the selection of biological drugs, the criteria for assessing treatment response, and how and when to terminate therapy in stable patients [102]. Recently, the European Academy of Allergy and Clinical Immunology (EAACI) [103], the GINA [12], and the GEMA [13] have proposed a series of recommendations. According to them, the establishment of a biological treatment should be based on phenotypic features (exacerbations, lung function impairment, comorbidities), some biomarker levels (peripheral blood eosinophils, FeNO, IgE, etc.), and clinical outcomes (exacerbations, lung function, control, comorbidities, quality of life and safety) [104,105].

As was previously mentioned, the T2 inflammatory phenotype is most commonly described in children with SA. This phenotype is characterized by a combination of pneumoallergen sensitization, elevated peripheral blood eosinophil levels, and/or elevated FeNO levels. Although these biomarkers are not closely correlated, more than 70% of patients with SA have one or more markers of T2 inflammation, and almost 40% of patients have two markers [106,107]. As was explained, three of these drugs, omalizumab, mepolizumab, and dupilumab, were approved by the FDA and the EMA for children aged ≥6 years. In comparison, the EMA approved benralizumab and reslizumab for patients aged ≥18 years and tezepelumab for adolescents aged ≥12 years. The latter is pending marketing in Spain at this article’s publication date. Thus, many patients would meet the criteria for initiating more than one biologic, making the initial choice difficult. In addition, biomarker levels, such as blood eosinophil counts and FeNO, are also dynamic and vary depending on the clinical situation, as well as by the influence of oral glucocorticoids. Therefore, ideally, biomarker determinations and studies should be performed in patients not receiving oral glucocorticoids and may even need to be delayed after receiving a course. Likewise, repeating biomarker levels when patients are in an exacerbation before starting oral glucocorticoids can also help to identify patients with T2 inflammation, as well as helping to change treatment if disease control is not achieved [108].

In essence, it is a matter of seeing what the patient´s phenotype (T2 or non-T2) and endotype (allergic or eosinophilic) are, bearing in mind that the current dichotomy between allergic and eosinophilic asthma classifications, although practical, is probably too simplistic. In fact, pathophysiology of eosinophil-mediated disease is quite similar in both classifications and also has some limitations in the pediatric setting [108]. The consideration of eosinophil as the predominant cell comes from findings obtained from both bronchoalveolar lavage or sputum samples, together with endobronchial-biopsy-derived tissue [109]. However, its relevance in contributing to the pathogenesis of asthma in pediatric patients seems to differ from that of adults, so their phenotypic characteristics should not be directly extrapolated [110]. In this regard, it has been observed that neither blood eosinophilia predicts greater airway eosinophilia (bronchoalveolar lavage) [111] nor do normal serum counts exclude the presence of pulmonary eosinophilia [112]. 

**Figure 3:**
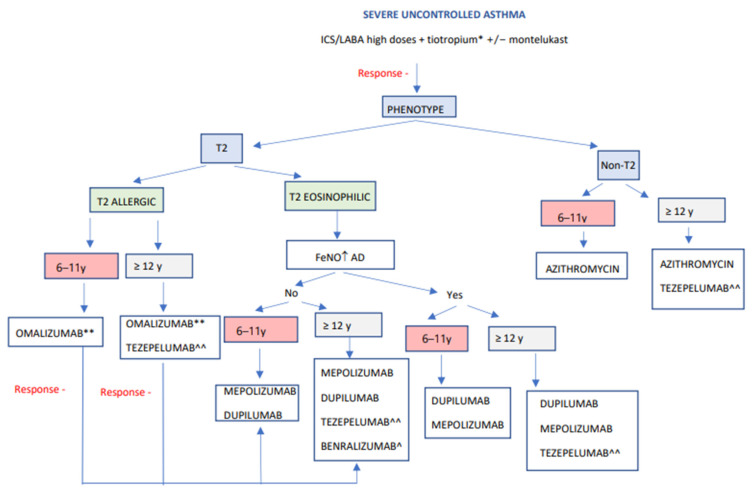
Algorithm for the evaluation and choice of a biological drug in children and adolescents with severe asthma. Modified from Valverde-Molina J. Curso en manejo del asma infantil. Módulo 4. Manejo del asma grave en el niño. Luzan 5. Health consulting SA. ISBN Módulo 4. 978-84-18420-90-0. ICS: inhaled glucocorticoids, LABA: long-acting bronchodilators, FeNO: fractional exhaled nitric oxide, AD: atopic dermatitis, y: years. * Considerer if lower lung function, higher reversibility, fixed obstruction, exacerbations. ** Considerer if atopic diseases coexist. ^ Considerer if eosinophils > 300 and age 18 years or older. ^^ Pending commercialization in Spain.

Taking into consideration all of the above, we present an algorithm (Figure 3) that allows for the selection of an appropriate biologic for a patient based on age and different biomarkers. This algorithm is centered on the phenotypic characterization of allergic asthma indicators, peripheral blood eosinophil counts, and FeNO levels [113]. Likewise, some authors suggest considering pulmonary function for choosing the most appropriate drug [22]. In this sense, when selecting a biological treatment for a compromised lung function child, a drug with improving lung function potential, for instance dupilumab, should be used. Other treatments with low effect on lung function, such as omalizumab and mepolizumab, should instead be avoided [114,115]. Another factor to consider is the presence of comorbidities that could also benefit from specific biological therapies. In the case of moderate-to-severe atopic dermatitis or eosinophilic esophagitis, along with T2 asthma, dupilumab could improve both diseases. In contrast, a patient with chronic spontaneous urticaria and allergic asthma would benefit significantly from receiving treatment with omalizumab. In addition, as previously mentioned, a new biologic therapy, tezepelumab, has recently been approved by the EMA, being found to be more effective in eosinophilic asthma. This treatment has also demonstrated its efficacy in patients with low levels of T2 inflammation biomarkers, offering great possibilities in this group of patients with less availability of biological drugs [116,117]. On the other hand, also in non-T2 patients, regular long-term use of azithromycin has been shown to reduce asthmatic exacerbations, independently of the inflammatory endotype. Still, it could be even more effective in people with airway colonization by Haemophilus influenza [118,119].

The decision-making process is complicated due to the absence of head-to-head comparative trials of biological treatment to determine whether the “best” option exists for a given patient. In this sense, a shared decision-making model could also be useful when choosing a drug for a particular patient [120]. Each patient and their family bring their unique beliefs, goals, and preferences to the discussion. Although all biological drugs require subcutaneous injections, the number and frequency of injections vary substantially from agent to agent. Many can be administered at home, and several have delivery device options. Side effect profiles differ among biologics and may influence patient preference. Some families may prefer older drugs with a longer history to recently approved drugs. Thus, a complete and open conversation about these aspects and drug attributes allows families to make decisions to improve treatment adherence and outcomes.

However, currently available studies suggest the possibility that new technologies combining both multiple data sources and artificial intelligence–machine learning, have the opportunity to offer a significant shift in asthma patient care and actual precision medicine in which therapeutic decisions are tailored to patient needs, clinical characteristics, specific markers of inflammation, comorbidities, and how these factors influence disease activity and progression [20].

## 5. Response Assessment

Once treatment with a biologic has been initiated, a close follow-up is essential to determine clinical response. In adults, although different proposals have been published, a consensus of experts has classified responses into three categories: super-responders, partial responders, and non-responders [121]. In this sense, a study with the participation of 81 experts from 24 countries using the Delphi methodology has reached a consensus on the definition of super-responders in SA [122]. A recent systematic review was the first study to evaluate the quality of the available evidence, including 13 reports using 17 response definitions. Among them, 16 (94.1%) had a high quality of evidence and 58.8% were based on the minimum clinically important difference, which could be insufficient in justifying the continuation of treatment in terms of cost-effectiveness. It would be necessary to include all those involved (organization, clinicians, patients) in order to develop universally acceptable criteria that would help to evaluate the efficacy of new therapies and improve clinical decision making and patient care [123].

With all the limitations involved in extrapolating studies from adults, and in the absence of similar reports in children, studies are needed to check whether this approach can be interesting and useful. In addition, we must highlight the relatively small number of children treated with biological drugs compared to adults, as well as the potential impact of the natural course of the disease in regard to the definition of responders/super-responders to treatment. In any case, there would be agreement on including four major domains when assessing response: severe flare-ups, use of oral corticosteroids, symptoms, and pulmonary function, to which should be added the assessment of quality of life and biomarkers. From a practical point of view, it is easy to identify responders (absence of severe attacks, symptom control, no oral corticosteroids, and normal lung function) and non-responders. The problem lies in cases of partial response and the need or not to modify the biological drug. Recently, a working group, COMSA, Working Group in the 3TR Consortium, was created that aims to provide information on the definitions of nonresponse and response to biological therapy for SA [124,125]. Some disease indicators, such as lung function and FeNO, may improve within 2 to 4 weeks after initiating treatment with dupilumab [95]. On the contrary, determining the impact on exacerbations requires a more extended observation period, which is not yet agreed upon [126].

A period of 4 to 6 months after the beginning of the treatment has been proposed to analyze the impact on exacerbations, lung function, asthma control, symptom burden, quality of life, and oral glucocorticoid use [22]. The occurrence of an exacerbation after initiation of biological therapy should not necessarily be considered a lack of response, as these drugs usually do not eliminate exacerbations. With some frequency, despite careful decision making based on phenotype, some patients may not experience optimal responses within 4 to 6 months. In those for whom the trial is extended for a few more months, this may allow for the detection of a more complete assessment of efficacy before considering switching to another biologic [22]. In the case of a partial or suboptimal response to any biological drug, different factors must be taken into consideration. These factors could be potentially responsible for this suboptimal response, such as incorrect identification of the endotype, the presence of associated diseases that have not been sufficiently treated, the use of insufficient dosage, the appearance of autoimmunity phenomena, and infections and/or changes in endotypes, among other factors [127]. Although it has not been carefully studied in children, reports in adults suggest that in patients who do not achieve control with a biological product, switching to a different one, generally with a different target, may contribute to a better clinical response. Before this, once an insufficient response has been found, a well-structured assessment of the possible causes of failure would be necessary. 

In its most recent update, the GINA considers that the evaluation should be performed at 3–4 months, including the following elements: asthma control (ACT, ACQ-5), frequency and severity of flare-ups, pulmonary function, management of comorbidities (if any), medication-related aspects (intensity, dose, adverse effects, accessibility), and patient satisfaction [12]. If the response to treatment is good, GINA proposes re-evaluations every 3–6 months, adapting the inhaled medication to the clinical evolution. If there is no good response, the case should be reviewed in depth, extending the complementary tests if necessary and evaluating the change to another biologic and/or establishing other treatments such as azithromycin or low-dose oral corticosteroids.

According to different studies, therapy with biological treatments would provide a 12.7–37% remission rate [42], although the concept of remission is currently under debate. In this way, there is a collective effort to reach a consensus on the definition of SA remission [128] such as the one currently underway in Spain (Foro de Asma SEPAR). It is trying to differentiate concepts such as clinical remission (which would include the absence of symptoms, exacerbations, and normalization or optimization of lung function with or without treatment) and complete remission, which would, in addition, include pathophysiological normalization (elimination of bronchial hyperresponsiveness and the reversibility of remodeling potentially possible by biological treatment) [129,130]. Long-term remission could be included as a therapeutic goal in asthma treatment studies, although it is possible that this remission is part of the natural history of the disease. Studies are needed to clarify this aspect [129].

## 6. Future Guidelines

Considering the different mechanisms underlying the pathogenesis of SA and response to treatment, guidelines based solely on clinical features compromise treatment success [131]. As noted, it is recognized that asthma in the pediatric population consists of different subtypes with variable clinical manifestations (phenotypes) [132]. Understanding the underlying biological mechanisms leading to these phenotypes (endotyping), diagnostic biomarkers and/or therapeutic options can be improved to allow for the personalization of treatment [133]. Therefore, a paradigm shift in the traditional way of diagnosing and treating asthma is necessary in the era of personalized medicine.

In recent decades, the development of molecular techniques has led to high-throughput analysis including different biological fields. This is called multilayer analysis, better known as omics-analysis (pharmacogenomics, epigenomics, transcriptomics, proteomics, metabolomics, and microbiome) [134]. Integrating omics data with clinical characteristics and laboratory parameters would allow for the definition of asthma endotypes and, thus, selecting the most appropriate therapy. Furthermore, integrating multiple omics approaches in childhood asthma has revealed new disease mechanisms and emerged as a viable option for moving toward precision medicine [135,136]. In this regard, in the last year, the role of different omics in response to asthma treatment has been extensively analyzed [137,138,139,140,141,142]. However, multi-omics projects in severe pediatric asthma pose a challenge regarding cost and computational and human resources required. Therefore, their success requires the coordination and collaboration of various research groups from different disciplines in an international multicenter approach [143]. Some of the successful multi-omics projects in the pediatric population are as follows: the Unbiased Biomarkers in Prediction of Respiratory Disease Outcomes (U-BIOPRED) [144] project, the Severe Asthma Research Program (SARP) [26,145], and the Systems Pharmacology Approach to Uncontrolled Pediatric Asthma (SysPharm PediA) cohort [134], among others, which have shown promising potential to reveal asthma phenotypes and delineate some possible endotypes [143].

### 6.1. Pharmacogenomics

Pharmacogenomics involves the study of genetic variation in the genome and its role in response to treatment. Most genetic studies of complex diseases such as asthma have focused on single-nucleotide polymorphisms (SNPs). Because of the patterns of polymorphisms, millions of genetic variants can be inferred from hundreds of thousands of SNPs. Thus, genome-wide association studies (GWAS) [146] can study genome-wide genetic variation without any prior hypothesis. In recent years, whole-genome sequencing (WGS) has emerged as a high-resolution method to study both common and rare genetic variations; however, although the WGS approach detects genetic variations not addressed by genome-wide genotyping arrays, its use remains to be limited due to economic reasons [147].

Most early pharmacogenomic studies of childhood asthma focused on populations of European descent [137,138]. Recently, two underrepresented populations with high asthma burden and treatment response failure have been analyzed [148]: African Americans from the Study of African Americans, Asthma, Genes and Environments (SAGE), and Hispanics/Latinos from the Genes–Environment and Admixture in Latin Americans (GALA II). These studies focused on the response to bronchodilator drugs [149] and the response to ICS, analyzing the presence/absence of severe exacerbations [150] as an outcome. Spear et al. [149] found a significant genome-wide association of the SNP rs73650726 at 9q21, and according to 1000 Genomes Project data, this SNP is only present in African/mestizo populations with a frequency of 9%, but not in Europeans or Asians. In addition, the trans-ethnic meta-GWAS in 2779 African American and Hispanic/Latino children and young adults identified the genome-wide association of three SNPs with response to SABA: rs7903366, rs7070958, and rs7081864 [151,152,153]. Likewise, it has been described that the combined effect of common and rare variants at three population-specific loci (1p13.2 and 11p14.1 in Mexicans and 19p13.2 in Africans) showed a significant genome-wide association for the bronchodilator test (BDT). In addition, two shared population loci (4q13.3 and 8q22.1) were also significantly associated with response to SABA [151]. Hernandez-Pacheco et al. [150] conducted a replication analysis of genomic regions associated with ICS response in previous GWAS focusing on Europeans and Asians and found a suggestive association for the rs5995653 SNP of the APOBEC3B-APOBEC3C intergenic region, which showed evidence of replication in 1697 European children with asthma. The A allele was consistently associated with improved response to ICS, measured as change in FEV_1_ after 6 weeks of treatment. The SNP rs62081416 near L3MBTL4-ARHGAP28 was associated with the response to ICS in children of mixed African race. L3MBTL4 and ARHGAP28 have been associated with pulmonary function after bronchodilator administration [154]. Likewise, Herrera et al. [155] identified in a cohort of Hispanic/Latino and African American asthmatic children and youth two significant genome-wide associations with susceptibility to severe exacerbations, including a novel locus located on chromosome 2p21 (rs4952375, odds ratio = 1.39; *p* = 3.8 × 10^−8^). The SNP rs1144986 (C5orf46) showed consistent effects for severe asthma exacerbations in all Hispanic/Latino subgroups but was not validated in non-Hispanics/Latinos. This SNP was associated with DPYSL3 DNA methylation and SCGB3A2169 gene expression levels [156].

Farzan et al. [157], within the multiethnic Pharmacogenomics in Childhood Asthma (PiCA) consortium, pooled data from ≥14,000 asthmatic children/young adults from 12 different countries in a cohort of >4000 asthmatic patients from 10 other studies (including patients of non-Hispanic European origin, two included Hispanic patients, one African American patient, and one East Asian patient) and studied the association between variation at the 17q21 locus, specifically at an SNP Rs7216389 strongly associated with pediatric asthma, exacerbations, and worse lung function, despite receiving ICS treatment. These and other asthma risk variants at 17q21 (rs4065275, rs12936231, rs7216389) increase ORMDL3 expression on CD4+ T cells, reducing interleukin-2 production. The authors concluded that rs7216389 appears to increase bronchial hyperresponsiveness and, thus, exacerbation rates in the pediatric population, suggesting that rs7216389 carriers may have a more severe form of asthma.

On the other hand, our group [158] was able to verify that of the 10 variants associated with asthmatic exacerbations despite treatment with ICS, only the SNP rs67026078, located within the intergenic region of CACNA2D3 and WNT5A, showed evidence of nominal replication after a meta-analysis of the European studies included in the replication, revealing itself as a new locus for exacerbations in European populations. Consistent with that published by the ENCODE project [159], the SNP rs67026078, with evidence of replication among Europeans, has been documented to be located within a histone H3 lysine 4 monomethylation tag (H3K4me1) in several tissues, including fetal lung fibroblasts and other fetal lung cells [160].

Shrine et al. [161] studied a cohort of European ancestry and identified new genetic variants associated with the risk of developing moderate and severe asthma that regulate mucin production, notably rs10905284 in GATA3, rs11603634 in the MUC5AC region, and rs560026225 near KIAA1109.

### 6.2. Epigenetics

An individual’s susceptibility to disease is not exclusively determined by the influence of the different genes and their combination but also by the environment, whose influence will be determined by the characteristics of the environmental factor itself and the time of life in which it interacts. In this genetic–environmental exposure relationship (allergens, viral and bacterial infections, etc.), the time of development of the immune system at which the interaction occurs is fundamental [162].

Epigenetics studies mechanisms that regulate gene expression without modifying the DNA sequence, such as DNA methylation (mDNA), microRNA (miRNA) regulation, and histone modifications. Today, epigenetic changes can be analyzed genome-wide (epigenomics) using high-throughput techniques. The most studied field is mDNA patterns, which consist of methylation of a cytosine base that occurs most frequently in regions where the cytosine is followed by a guanine in the 5′–3′ direction (CpG sites) [163]. 

Two epigenome-wide association studies (EWAS) by Wang et al. [164,165] analyzed the association of CpG site methylation status with treatment response in childhood asthma. In the first [164], a relative hypomethylation of the CpG site cg00066816 nearIL12B showed a protective effect against severe exacerbations in a meta-analysis of non-Hispanic whites from the Childhood Asthma Control Program (CAMP); Europeans from the Children, Allergy, Milieu, Stockholm, Epidemiology (BAMSE) study; and Hispanics/Latinos from the Genetic Epidemiology of Asthma in Costa Rica Study (GACRS). Hypomethylation of cg00066816 and the absence of severe exacerbations were shown to be specific to patients treated with ICS. They found 13 CpG sites significantly associated with the absence of oral corticosteroid (OCS) use in a meta-analysis among non-Hispanic whites from CAMP and Costa Ricans from GACRS. An interaction analysis identified that hypermethylation of cg04256470 near CORT-CENPS was associated with no OCS use, specifically in patients treated with ICS. In the second [165], relative hypermethylation of cg27254601 of BOLA2 was associated with improved lung function and increased expression of BOLA2, which encodes a protein involved in the maturation process of iron- and sulfur-containing proteins and whose intronic variants have been associated with eosinophil levels and lung function. In addition, hypermethylation in PBC of the OTX2 gene was associated with an improvement in FEV_1_ after ICS treatment. 

In this regard, our group [166] studied in the SysPharm PediA cohort a group of European children from four different countries diagnosed with moderate and severe asthma, observing that CpG cg12835256 (PLA2G12A gene) was significantly associated with FeNO, and three CpGs were associated with BDT, highlighting CpG cg26203256 (ADD3-AS1 gene). These genes are key in regulating type 2 inflammatory processes, bronchial hyperreactivity, and immunoglobulin E levels. Twelve and four differentially methylated regions (DMRs) associated with FeNO and BDT, respectively, were identified. Higher methylation could imply lower gene expression, type 2 inflammation, BDT, and FeNO. These findings can allow for the discovery of new biomarkers in managing moderate–severe asthma.

Kho et al. [167] evaluated the role of 754 circulating miRNAs on OCS use in serum samples from non-Hispanic white children with CAMP asthma before initiating ICS treatment. They found that 12 miRNAs were associated with the risk of moderate–severe asthmatic exacerbations despite ICS treatment, with miR-206 showing the strongest association. Furthermore, these miRNAs were included in a combined predictive model of asthmatic exacerbations based on clinical characteristics, with three of these circulating miRNAs (miR-206, miR-146b-5p, and miR-720) and better-predicted asthma exacerbations being found in children treated with ICS than a model that only included clinical parameters.

### 6.3. Transcriptomics

Transcriptomics studies all RNA transcripts by high-throughput methods such as RNA sequencing (RNA-seq) or microarrays. Most transcriptomic studies of treatment response in pediatric asthma have focused on ICS. Microarray analysis of peripheral blood mononuclear cells (PBMC) from asthmatic children revealed that patients with poor disease control showed specific transcriptomic patterns associated with ICS signaling and immune response compared to other asthmatic children [168]. In addition, Qui et al. [169] in non-Hispanic white CAMP children treated with ICS analyzed gene expression networks in B-cell lines, and good responders to treatment showed enrichment in immune response and ICS-induced proapoptosis. In contrast, poor responders showed enrichment in anti-apoptosis pathways. Two transcription factors (T.F.s), NFKB1 and JUN, showed a remarkable differential regulation between the two groups. They assessed the effect of these T.F.s on the expression of nine downstream genes by silencing T.F.s. They found that CEBPD (regulated by NFKB1) was overexpressed in good responders, whereas TMEM53 (regulated by JUN) showed the opposite effect.

McGeachie et al. [170] performed a multi-omics analysis in 104 non-Hispanic white children with CAMP asthma treated with budesonide. Treatment response was assessed by steroid response endophenotype (SRE), a composite phenotype that predicted response to ICS. The SRE index, genome-wide genotyping data, and lymphoblastoid cell response to dexamethasone were integrated to construct an ICS response network. This systems biology approach identified seven genes associated with ICS response, four of which were selected for in vitro validation analysis. Knockdown of one of these genes, FAM129A, reduced the response to dexamethasone (*p*-value < 0.001) in lung epithelial cells. Interestingly, this gene codes for a protein involved in an apoptosis pathway and thus could potentiate the anti-inflammatory effect of ICS [171].

Katayama et al. [172] performed a transcriptomic study evaluating the response to anti-leukotrienes (ALT) in 107 children aged 6 to 48 months. Weighted gene co-expression network analysis (WGCNA) was performed to identify subsets of strongly correlated genes (modules) involved in response to ALTs. WGCNA of gene expression in leukocytes identified a module of 145 coregulated genes correlated with wheezy bronchitis. It showed a positive correlation with lung function and ATL treatment, a negative correlation with vitamin D levels at seven years, and the number of exacerbations during follow-up. This module also predicted the need for future ALT treatment. The gene with the strongest association with ATL treatment was TRIM22, which encodes a protein involved in the antiviral response regulated by an interferon pathway [173].

### 6.4. Metabolomics

Metabolomics aims to profile biological samples’ complete metabolite composition (metabolome). High-throughput analytical techniques, such as mass spectrophotometry, nuclear magnetic resonance, or spectroscopic methods, allow metabolites to be characterized in invasive samples such as blood and non-invasive ones such as exhaled air (breathomics) and have been successfully applied for asthma phenotyping [174,175], helping to understand the response to treatment.

Kelly et al. [176] evaluated the interaction of age and serum metabolites with BDT in asthmatic children. Thirty-nine metabolites, mainly lipids, showed a nominal interaction with age on BDT, with the strongest interaction observed being that of 2-hydroxyglutarate. In addition, they also performed a multi-omics study of lung function in Hispanic/Latino children with asthma [177]. After adjusting for confounding factors, they found four transcriptomic modules and five groups of metabolites related to lung function, with interactions found among seven of them. A transcriptomic module enriched in asthma-related miRNAs was associated with BDT and lipid metabolomics module. ORMDL3, a widely studied gene in pediatric asthma that plays a role in sphingolipid biosynthesis [178], was identified as one of these transcriptomic modules. Based on genotyping data, SNP rs8079416 within ORMDL3 was an eQTL (expression quantitative trait loci, genomic loci that contribute to variation in mRNA or protein expression levels) in this population. It was also associated with 165 of the 537 lipids included in the metabolomic module. Therefore, it is likely that the relationship between ORMDL3 expression, the microRNA regulatory motif, and sphingolipid metabolism plays a role in BDT.

### 6.5. Microbiome

The composition of microbial communities, or microbiota, is conditioned by intrinsic and extrinsic factors. Microbial exposure is essential for developing the immune system, and differential changes in the microbiota have been associated with allergic diseases [179]. Dysbiosis in microbial communities and bacterial pathogens at different sites in the body have been linked to the development of allergic diseases, probably due to dysregulation of the host immune response [180]. To date, the bacterial microbiome has been extensively studied by targeted sequencing of the 16S ribosomal RNA (16S rRNA) gene, a prokaryotic marker whose hypervariable regions contribute to the taxonomic classification of bacteria at the genus level [181]. 

The airway microbiome is altered in asthmatic patients and differs from immune response models, especially in the levels of T2 inflammation [182]. Clinical studies have demonstrated a relationship between alterations in the pulmonary microbiota and various asthma phenotypes [183], and it has been described that a reduction in bacterial diversity could influence the inflammatory phenotypes of asthma [184]. Likewise, it has been described that dysbiotic communities can contribute substantially to the course and severity of the disease, and a relationship has been observed between airway microbial dysbiosis and progression, exacerbations, and response to treatment [185]. In this regard, Zhou et al. [186] studied the nasal bacterial microbiome by 16S rRNA sequencing in 214 European children with mild–moderate asthma treated with low-dose ICS. Children with a nasal microbiome dominated by Corynebacterium and Dolosigranulum during periods of good disease control had fewer episodes of loss of control and more time to develop at least two exacerbations compared to children with a microbiota dominated by Staphylococcus, Streptococcus, or Moraxella. In addition, during the first episode of loss of control, Streptococcus was the most prevalent dominant genus in the nasal microbiome. During the loss of control, total bacterial richness and load were significantly higher than at the time of good control. In addition, a higher relative abundance of Corynebacterium was associated with a lower risk of exacerbations requiring OCS. Finally, the dominant genus shifts from Corynebacterium + Dolosigranulum to Moraxella was associated with an increased risk of OCS use. Corynebacterium is the most abundant commensal bacterium in the nasal microbiome of healthy individuals, and its relative abundance decreases in asthmatic patients [187]. In addition, Moraxella, Streptococcus, and Hemophilus are more frequent bacterial pathogens in asthmatic patients’ nasal microbiomes than in healthy individuals, which could lead to activation of the Th2 inflammatory response. Likewise, Moraxella has also been associated with an increased risk of asthmatic exacerbations [181,188].

Within the Genomics and Metagenomics of Asthma Severity (GEMAS) project [189], which included 250 asthmatic patients treated with ICS, our group studied the association between the salivary, pharyngeal, and nasal microbiome with asthma exacerbations, wherein it was observed that, in nasal and saliva samples, those who had presented moderate or severe exacerbations (use of OCS and/or hospital admission) had less bacterial diversity than those who did not present exacerbations [190]. Exacerbations accounted for 8% to 9% of the interindividual variation in the salivary and nasal microbiomes, respectively. Three, four, and eleven bacterial genera of the salivary, pharyngeal, and nasal microbiomes were differentially abundant between groups, respectively. Integration of clinical, genetic, and microbiome data showed good discrimination in terms of identifying the likelihood of asthmatic exacerbations despite ICS treatment, concluding that the diversity and composition of the upper airway microbiome are associated with asthmatic exacerbations. Therefore, the salivary microbiome has potential application as a biomarker of exacerbations in asthmatic patients. 

In recent decades, the role of the gut microbiome and its metabolites in the pathogenesis of asthma has also been studied. In this regard, Van Nimwegen et al. [191], in a prospective cohort in the Netherlands (KOALA Study), analyzed the intestinal microbiome, finding an association between colonization at one month of life by Clostridium difficile species (phylum Firmicutes) with the presence of wheezing and asthma at the age of 6–7 years. Abrahamsson et al. [192], in a cohort from Sweden, found a relationship between a lower diversity in the intestinal microbiome in the first month of life and the development of asthma at seven years of age. Arrieta et al. [193], in a Canadian cohort (CHILD Study), found that children at risk of developing asthma had lower relative amounts of bacteria of the genera Faecalibacterium, Lachnospira Veillonella, and Rothia at early ages. These results increased the likelihood of developing future diagnostic tools and microbial-based treatments, potentially in probiotics, in order to prevent the development of asthma and other allergic diseases in the pediatric population. In this regard, our group [194] analyzed the gastrointestinal microbiome as a potential marker to discriminate between uncontrolled and controlled asthma in children in a cohort of 143 children with moderate–severe asthma in the SysPharmPediA study. Machine learning, specifically recursive ensemble feature selection (REFS), found that controlled and uncontrolled asthmatic children could be differentiated based on their gastrointestinal microbiome, identifying a set of taxa, including Hemophilus and Veillonella, that allowed for classification with an average accuracy of 81% (in saliva) and 86% (in stool). These results show an association between asthma control and the gastrointestinal microbiome, which suggests that the gastrointestinal microbiome may be a possible biomarker of response to treatment and thus help improve disease control in children.

However, although omics studies have provided information on the biological mechanisms involved in treatment response, further studies are required to refine predictive markers with clinical relevance. A better understanding and identification of the molecular mechanisms underlying the different phenotypes within pediatric asthma, especially those with a higher tendency to exacerbate and worse response to conventional treatment, could lead to better classification and management of patients and the identification of new therapeutic targets. The integration of clinical characteristics with multiple omics data is a very promising avenue that will facilitate the management of the disease since it will contribute to providing genetic and non-genetic biomarkers that will allow for the identification and classification of the different phenotypes of asthmatic patients with poor or partial response to the usual treatment with ICS.

## 7. New Therapeutic Targets

Ongoing research into new mAbs focuses on new therapeutic targets or cytokines, including alarmins [30,42,43,195]. One example is astegolamib, an anti-SAT2 (IL-33 associated receptor) that could have a role in patients with a low T2 phenotype [196]. In a phase II study including 296 patients with moderate–severe asthma, itepekimab (a human IgG4P mAb against IL-33) was analyzed. In this study, four groups randomly received itepekimab 300 mg S.Q., dupilumab 300 mg S.Q., itepekimab + dupilumab 300 mg S.Q., or placebo every 2 weeks for 12 weeks. After analysis, a loss of control in 22% of the itepekimab group, 19% of the dupilumab group, 27% of both, and 41% of the placebo group was described, concluding that itekinumab + dupilumab was not superior to both separately in asthma control. The incidence of adverse effects was similar in all groups [197]. A clinical trial on the efficacy and safety in uncontrolled moderate–severe asthma of tozorakimab (MEDI3506) [198] (a human IgG1 anti-IL-33 antibody that inhibits IL-33 signaling through ST2 and RAGE/EGFR to reduce inflammation and epithelial dysfunctions) (FRONTIER-3) (NCT04570657) [199] has recently been completed.

IL-23 promotes Th17 cell proliferation, neutrophil recruitment, and T2 cytokine production. A 24-week, multicenter, randomized, double-blind, placebo-controlled, multicenter, phase IIA study, which included 214 patients with moderate–severe asthma, evaluated the efficacy and safety of 90 mg S.Q. of risankizumab every 4 weeks. This is a mAb acting on IL-23. According to this study, during the analysis, time to asthma worsening was shorter, as well as and the annualized rate of asthma worsening being higher when risankizumab was compared with the placebo [200]. Other antineutrophilic therapeutic targets, such as anti-IL17 and anti-CXCR2, have not shown improvement in this phenotype. A possible explanation to these findings could be that this type of inflammation is secondary to microbial dysbiosis [30].

TL1A is a member of the TNF superfamily of ligands. It binds to the DR3 receptor, which is constitutively expressed at low levels on T, B, and N.K. cell surfaces. This binding is important for sustained pathological T2 responses. Its blockade with neutralizing antibodies reduced inflammation and levels of IL-4, IL-5, and IL-13 cytokines in a murine ovalbumin-induced asthma model. A new human antibody (C03V), which binds to an epitope of TL1A, allows for a very potent and selective neutralization of DR3 signaling and is potentially useful for the treatment of diseases involving TL1A dysregulation, including diseases with a fibrotic component, such as asthma [201]. Currently, a 16-week, randomized, double-blind, placebo-controlled, parallel-group study has just been completed, evaluating the efficacy and safety of TEV-48574 (fully human IgG1 monoclonal antibody specific for TL1A) in adults with uncontrolled low T2 or non-T2 G.A. (NCT04545385) [202].

FB704A (a fully human monoclonal antibody that inhibits the IL-6/IL-6R signaling pathway by neutralizing IL-6) may reduce BHR, as well as airway T1, T2, and T17 inflammatory responses, thus having the potential to improve symptoms of SA (with elevated neutrophils) and severe mixed granulocytic asthma. A phase IIA, randomized, placebo-controlled, double-blind, study is underway to evaluate the safety, tolerability, pharmacokinetics, and clinical activity of multiple intravenous doses of FB704A in adults with SA (NCT05018299) [203].

Another future strategy could be the design of new forms of administration that would improve therapeutic concordance, effectiveness, and side effects by reducing systemic impact. An inhaled anti-TSLP mAb, ecleralimab, is currently under investigation. A phase II study showed that ecleralimab significantly attenuated allergen-induced bronchoconstriction and airway inflammation and was safe in adult patients with mild asthma, which represents a promising route of administration in the future [204].

## 8. Conclusions

The appearance of new biological treatments for pediatric patients with UCSA has meant a paradigm shift and an improvement in the personalized management of the disease. Still, more studies are needed to provide answers to different aspects that have not yet been clarified [22,41]. These studies should include ideal candidate for different biologics, predictors, markers of response, duration of treatment, criteria for discontinuation, effectiveness, and safety in the pediatric population. Comparative studies between different mAbs, new biomarkers, and biologics in non-T2 asthma, as well as studies directed toward modifying the natural history of the disease, are also needed. 

## Figures and Tables

**Table 1:** Monoclonal antibodies in the pediatric population with severe asthma.

Name	Target	Mechanism of Action	Authorized (Age)		Indication	Dosage S.Q.	Comorbidities Treatable	Predictors Response	Clinical Outcomes					Adverse Effects
			FDA	EMA					E	C	P.F.	QoL	OCS	
OMALIZUMAB	IgE	Circulating IgE binding preventing receptor binding FcεR.I. in mast cells, basophils, and dendritic plasmacytoid cells; FcεRII in dendritic plasmacytoid cells and Eos. Reduction of free IgE and downregulation of receptor expression.	≥6	≥6	Uncontrolled allergic SA with sensitization to perennial pneumoallergens and in range according to weight and IgE.	By weight and total IgE: FDA: 75–375 mg IgE (KU/L): 6–11 y: 30–1300 mg. : 30–700 mg. EMA: 75–600 mg IgE (KU/L): ≥6 y: 30–1300 mg/2–4 w. Prefilled syringe. Home administration.	Idiopathic chronic urticaria. Nasal polyposis.	Eos ≥ 260/μL FeNO > 20 ppb	↓	↑	=↑	↑	↓	Local reaction. Headache. Fever (6–12 years). Anaphylaxis (very rare).
MEPOLIZUMAB	IL-5	Circulating IL-5 binding prevents binding to the α-receptor. Eos reduction.	≥6	≥6	SA uncontrolled and Eos ≥ 150/μL or ≥300/μL in the last year.	6–11 y: 40 mg. ≥12 y: 100 mg/4 w. Prefilled syringe or autoinjector (pen). Home administration.	Nasal polyposis. EGPA. HES.	↑ Eos ↑ E Nasal polyposis OCS	↓	↑	↑	↑	↓	Local reaction. Headache. Nasal congestion. Anaphylaxis (very rare).
BENRALIZUMAB	IL-5Rα	Binding to IL-5Rα Rapid apoptosis of Eos by cytotoxicity mechanism.	≥12	No	SA uncontrolled and Eos ≥ 150/μL or ≥300/μL in the last year.	30 mg/8 w (first 3 doses every four w). Prefilled syringe or autoinjector (pen). Home administration.		↑ Eos ↑ E Nasal polyposis OCS ↓ PF	↓	↑	↑	↑	↓	Local reaction. Headache. Pharyngitis. Anaphylaxis (very rare).
DUPILUMAB	IL-4Rα	Binding to IL-4Rα blocking IL-4/IL-13 signaling. T2 inflammatory pathway downregulation. Prevent Eos extravasation to tissues.	≥6	≥12	SA uncontrolled and Eos ≥ 150/μL and ≤1500/μL and/or FeNO ≥ 25 ppb and/or need for OCS.	FDA: 6–11 y ≤ 30 kg:100 mg/2 w or 300 mg/4 w. 6–11 y > 30 kg: 200 mg/2 w or 300 mg/4 w. FDA and EMA ≥ 12 y: 200 mg/2 w (first dose 400 mg). Prefilled syringe or autoinjector (pen). Home administration.	AD. Nasal polyposis. EEo.	↑ Eos ↑ FeNO	↓	↑	↑	↑	↓	Local reaction. Transient elevation of eosinophilia. EGPA (very rare). Anaphylaxis (very rare).
TEZEPELUMAB	TSLP	Binding to circulating TSLP prevents receptor binding. Acts at high levels of the inflammatory cascade.	≥12	≥12	SA T2 or non-T2 with exacerbations.	210 mg/4 w. Prefilled syringe or autoinjector (pen).		↑ Eos ↑ FeNO T2 low	↓	↑	↑	↑	=	Local reaction. Pharyngitis. Arthralgias. Lumbar pain. Nasal congestion. Anaphylaxis (very rare).

IL-5Rα receptor α-subunit, IL-4Rα: IL-4 receptor α-subunit, SA: severe asthma, S.Q: subcutaneous, FDA: Food and Drug Administration, EMA: European Medicines Agency, EGPA: eosinophilic granulomatosis with polyangiitis, HES: hypereosinophilic syndrome, AD: atopic dermatitis, EEo: eosinophilic esophagitis, FeNO: fractional exhaled nitric oxide, ppb: parts per billion, Eos: peripheral blood eosinophils, OCS: oral corticosteroids, PF: pulmonary function, E: exacerbations, C: control, QoL: quality of life, w: week, y: year.

## Data Availability

Not applicable.
